# Use of biochemical and imaging criteria for selecting patients for prostate biopsy in recurrence risk assessment post-HIFU therapy

**DOI:** 10.1007/s00345-025-05529-0

**Published:** 2025-03-12

**Authors:** Tarek Ajami, Adam Williams, Jonathan T. Ryan, Nachiketh Soodana Prakash, Archan Khandekar, Keerthana Sureshkumar, Chad R. Ritch, Mark L. Gonzalgo, Sanoj Punnen, Dipen J. Parekh, Bruno Nahar

**Affiliations:** 1 Desai Sethi Urology Institute, University of Miami, Miller School of Medicine, Miami, FL USA; 2 Department of Urology, Hospital Clinic de Barcelona, University of Barcelona, Barcelona, Spain; 3 Desai Sethi Urology Institute, 1120 NW 14th St #2107, 15th Floor, Miami, FL 33136 USA

**Keywords:** Prostate cancer, High-Intensity focused ultrasound, MRI, PSA kinetics

## Abstract

**Purpose:**

Despite the growing adoption of HIFU treatment for localized prostate cancer (PC), standardized criteria for evaluating success and predicting recurrence remain undefined. Herein, we analyze the predictive value of noninvasive tools such as PSA dynamics and MRI to determine recurrence.

**Methods:**

We identified from our HIFU therapy prospective registry patients who developed biopsy-proven recurrence, between 2016 and 2023. Clinically significant recurrence (CS-R) was defined as the presence of GG 2 or greater on control biopsy. Different modalities of PSA kinetics were analyzed and determinants for recurrence were based on either PSA or MRI criteria (PIRADS > 3). Sensitivity, specificity, PPV, and NPV were estimated based on single or combined criteria.

**Results:**

92 patients were included in the study. A total of 17(18%) patients had CS-R. Those patients presented higher PSA velocity (*p* < 0.001) and a higher proportion of PSA above nadir + 1 at 12 months (*p* = 0.001). Static PSA measurement and % of PSA drop were not associated with recurrence. Follow-up based on a combination of PSA parameters (PSA below nadir + 1) and MRI criteria had higher sensitivity (88%) and negative predictive value (96%) in detecting post-treatment recurrence. Limitation of the study included limited number of patients and a relatively short follow up period.

**Conclusions:**

Post-HIFU recurrence surveillance through dynamic PSA monitoring shows better predictive value. Implementing ‘for cause’ surveillance biopsies guided by dynamic PSA changes along with mpMRI appears to enhance the detection of recurrences without missing a significant number of cases.

## Introduction

Focal therapy (FT) with High-intensity focal ultrasound (HIFU) energy is currently being offered as an alternative to whole gland treatment for selected cases of localized prostate cancer (PC), driven by the goal of optimizing functional outcomes without jeopardizing oncological control. Oncologic outcomes of FT from prostpective cohorts are favorable [1–3], but there is still an initial risk of recurrence after treatment and, thereby, a close follow up is warranted.

Currently, HIFU treatment is considered as an intermediate therapeutic tool between active surveillance (AS) and whole gland treatment. The adoption of MRI and biomarkers into the field of AS have pushed towards a risk adapted approach in follow-up for low risk prostate cancer, decreasing thereby the burden of repeated biopsies. Promising results from prospective studies from AS cohorts have paved the path towards a less invasive followup in patients undergoing focal therapy. In fact, few focal therapy series have analysed the role of MRI and its interpretability in post-ablation setting [4].

Despite the growing adoption of FT, standardized protocols for follow-up to define biochemical recurrence and treatment efficacy are still lacking. Initially, the Phoenix criteria were adopted for post-HIFU follow-up in the context of whole gland ablation. However, with the shift towards focal HIFU therapy, the usage of the criteria has declined [5]. Additionally, consensus on the best definition of recurrence has yet to be established; recent prospective studies uniformly rely on protocol-mandated post-ablation biopsies to identify treatment failure [6–8]. In contrast, other authors advocate for a “for cause” MRI and/or biopsy in case of a rising PSA after treatment [1, 2].

Herein, we examined the predictive performance of PSA dynamics and MRI in triggering post-ablation biopsies within a prospective cohort of patients treated with HIFU for localized prostate cancer. We also analysed different strategies for follow up and their outcomes in reducing the need for post ablation biopsies.

## Patients and methods

This prospective single center study at University of Miami was approved by institutional review board (IRB# 20160385) [3, 9]. Patients included in the study were treatment-naïve patients diagnosed with localized PC between January 2016 and January 2023 and had at least 12 months of follow-up.

All patients had an initial 12-core systematic prostatic biopsy in addition to 2 targeted cores for any suspicious lesions identified on multi-parametric MRI. Patients with localized PC and eligible for focal or hemi gland ablation were included. Our study included all Gleason grades and risk classification was based on NCCN risk groups. However, very low-risk patients were steered towards active surveillance, and high-risk patients were eligible for HIFU if they presented an MRI-identifiable lesion and no more than 2 cores with > GG3 cancer. All patients had baseline PSA, MRI, and biopsy in addition to an assessment of urinary and sexual functions. HIFU was performed using Ablatherm^®^ until 2019 (EDAP) and Focal One^®^ (EDAP) from 2020 to 2023.

Follow-up protocol included PSA measurements every 3 months for 2 years, followed by biannual measurements. Functional outcomes were assessed at 3, 6, 9 and 12 months. Per study protocol, all patients were required to have an MRI and subsequent MRI/US fusion biopsy within 12 months post-treatment. Patients with negative MRIs were advised to have a systematic biopsy. Recurrences were classified by location within the treatment field (infield or outfield) and by grade (clinically significant (CS) if *≥* GG2 is detected or non-significant when GG1 is obtained) [3].

### Statistical methods

Descriptive statistics and frequency tables were used to summarize the data. Pearson’s chi-square tests (or Fisher’s exact test) were used for categorical variables. Several postoperative PSA parameters were tested, including PSA nadir, PSA reduction, and PSA velocity (PSAV) during the first year, calculated either by algorithmic method or linear regression [10]. PSAV by arithmetic method is calculated as the rate of change of PSA from month 3 to month 12, whereas linear regression represents the slope of a linear equation model. We tested the Phoenix (PSA nadir + 2) [11] and Huber criteria (PSA above nadir + 1) as definitions for recurrence [12]. A multivariate logistic regression was performed to account for potential confounders in identifying predictors for CS recurrence. In addition, several strategies for follow up were desgined based on PSA or MRI criteria. Diagnostic accuracy measures such as sensitivity, specificity, and negative predictive values were derived to obtain the optimal probability of detecting recurrence using various models: PSA metrics, MRI, or a combination of both criteria (sequential or combined). These were then compared to the ground truth established by the protocol biopsy. Since our data indicated a low incidence of cancer in patients with PSA < 1 (only 2 patients in this group had cancer; none with GG2 or higher), those who refused a biopsy but had a stable PSA < 1 were considered cancer-free.

Statistical analyses were performed using SPSS version 29. A p-value < 0.05 indicated statistical significance.

## Results

### Baseline characteristics

Between 2016 and 2023, 113 patients underwent FT for prostate cancer. 92 (81%) patients completed at least one year of follow-up and were included in the study. The majority (75%) had GG2 or higher. 82(89%) patients had a post-treatment MRI within 12 months after treatment. 71(77%) patients completed their post-ablation biopsies. Table 1 lists the cohort’s baseline pathologic and radiologic characteristics.

**Table 1:** Baseline, operative and postoperative characteristics of patients who underwent primary HIFU

		*n* = 113 (IQR)
**Baseline Characteristics**		
Age		68 (62–72)
Baseline PSA		5.7 (4.3–7.6)
Prostate volume		41 (30–61)
PSA density		0.14 (0.08–0.19)
Ablation		
Focal		15 (14%)
Hemigland		98 (86%)
GG	1	28(25%)
	2	52 (45%)
	3	21 (18.4%)
	4	6 (5.3%)
	5	6 (5.3%)
Pre-treatment MRI	No lesion	12 (11)
	PIRADS 3	13 (12)
	PIRADS 4	55 (51)
	PIRADS 5	25 (26)
Lesion locations		
TZ		17 (18)
PZ		78 (82)
Combined TURP or HOLEP (%)		26 (23%)
NCCN group		
Low risk		24 (21%)
Intermediate favorable		56 (50%)
Intermediate unfavorable		21 (19%)
High risk		12 (10%)
**Postoperative Data** (***n* = 92)**		
Median nadir PSA		1 (0.45–2.2)
% PSA reduction at 3 months		75 (52–90)
% PSA reduction at 6 months		77 (56–89)
% PSA reduction at 12 months		64 (48–90)
Post ablation MRI (*n* = 82)	Negative	55 (67.1%)
	PIRADS 3	11 (13.4%)
	PIRADS 4–5	16 (19.5%)
Per protocol Biopsy (*n* = 71)	Benign	37 (52.1%)
	GG1	18 (25.4%)
	GG2	6 (8.5%)
	> GG3	10 (14%)

### Pathologic and radiologic results

The overall rate of positive biopsies was 37%, of which 16 (17%) patients were clinically significant prostate cancer (csPCa). Among these, 11 (12%) occurred within the treatment area (infield).

Among the patients who underwent a post-treatment MRI, 55 (67.1%) had a negative MRI result, 11 (13%) showed PIRADS 3 lesions, and 16 (19%) were found to have lesions classified as PIRADS 4 or 5. For those with suspicious MRI lesions, 13 (54%) were infield, and 10 (41%) were outfield. As expected, none of the PIRADS 3 lesions were associated with csPCa, whereas 53% of those with PIRADS 4 (50%) or 5 (57%) lesions were found to have csPCa. Of those with a negative MRI, 41 (74%) patients underwent subsequent biopsy: 26 (63%) were negative, 9 (22%) had GG1 disease, and 6 (15%) had clinically significant PCa (csPCa).

### Predictors of recurrence

**Fig. 1:**
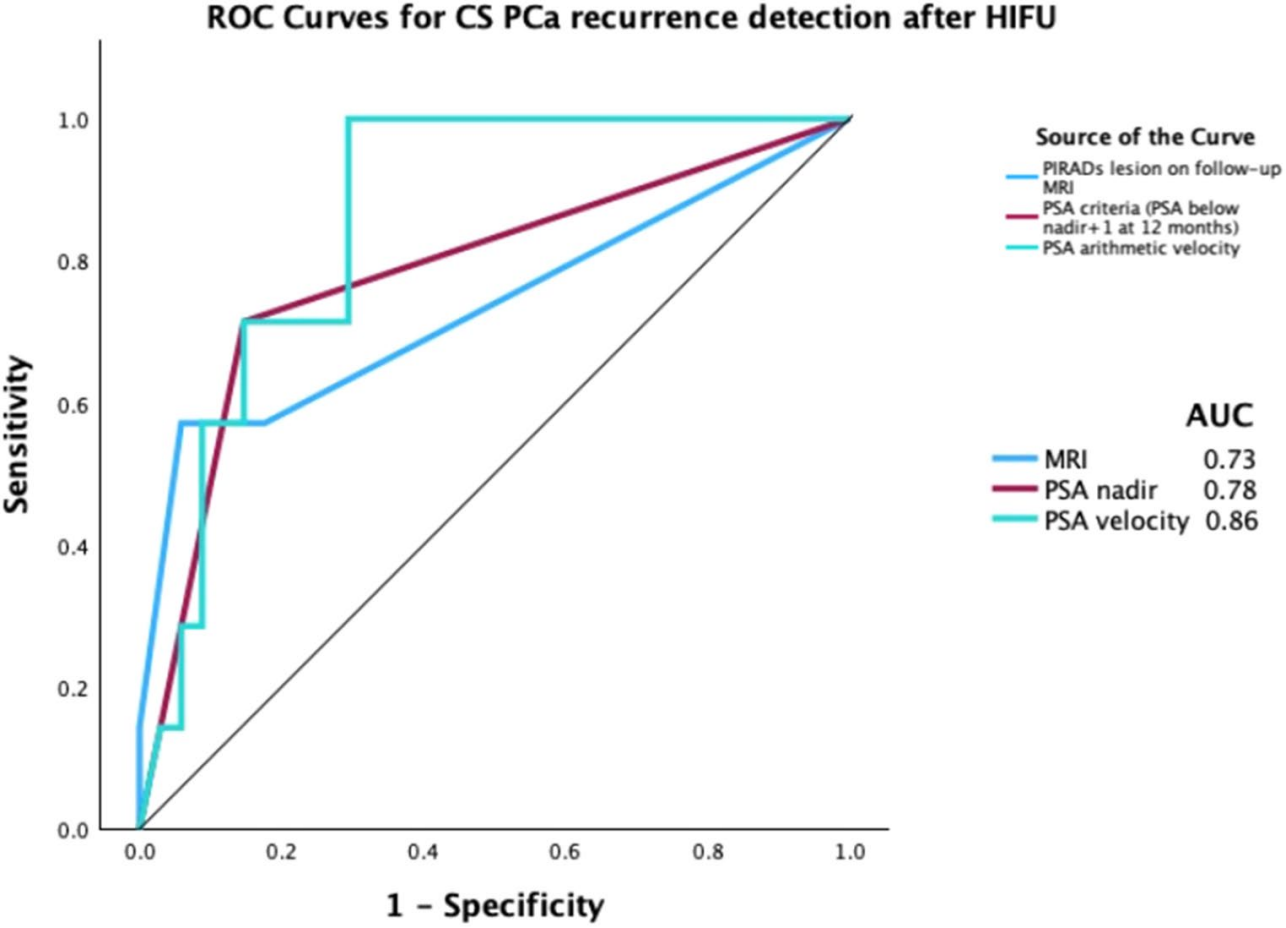
The ROC curves of PSA parameters MRI findings (PIRADS > 3) in predicting prostate cancer recurrence after HIFU

Preoperative median PSA was 5.7 ng/ml. Median nadir PSA was 1 ng/ml, with a median time to nadir of 6 months. The median percentage of PSA drop from baseline was 75%, 77% and 64% at 3, 6 and 12 months, respectively (Table 1). 28 (30%) patients had PSA at 12 months < 1 ng/ml. 17 (60%) patients underwent protocol biopsy: 2 patients had GG1 disease, and no csPCa was detected. Patients with clinically significant recurrence had significantly higher PSA velocity measured by either algorithmic or linear regression equation compared to non-recurrent patients and non-clinically significant recurrences, as well as higher proportion of patients with PSA at 12 months greater than nadir + 1 (Huber criteria). Notably, neither nadir PSA nor %PSA reduction were different across the recurrence groups (Table 2). Multivariate logisitic regression showed that the presence of non-equivocal lesion on MRI (PIRADS 4–5) or PSA > nadir + 1 were independently associated with higher likelihood of harboring CS recurrence (Table 3), regardless of initial NCCN risk group. The AUC for detecting recurrence using MRI with PIRADS 4 or 5, PSA nadir + 1, and PSA velocity was 0.73, 0.78 and 0.86, respectively (Fig. 1). We further analyzed the independent and combined PSA and MRI diagnostic performance to detect recurrences, as detailed in Table 4. When used independently, the diagnostic performance of PSA criteria based on the Huber definition (strategy A) for recurrence and MRI with PIRADS 4 or 5 (strategy B) had a sensitivity of 69% and 50% and specificity of 86% and 89%, respectively. Utilizing MRI following a positive PSA criterion increased the specificity to 97%. This approach would avoid 79% of unnecessary biopsies while missing 12% of recurrences. Utilizing both a PSA criteria and/or positive MRI (PIRADS 4 or 5) yielded a sensitivity and specificity of 88% and 78%, respectively, and avoided 64% of unnecessary post-treatment biopsies.

**Table 2:** Comparison of post-treatment PSA parameters and MRI features based on recurrence patterns

		CS recurrence (*n* = 16)	non CS recurrence (*n* = 18)	No recurrence (*n* = 58)	*P* value of CS recurrence vs. others	*P* value of any recurrence vs. no recurrence
PSA nadir (ng/ml)		1.54 (0.7–4.1)	1.95 (0.9–3.1)	0.7 (0.33–1.64)	0.74	0.113
PSA nadir > 2 (%)		6/16 (40%)	9/18 (50%)	11/58 (20%)	0.548	0.019
%PSA drop at 3 months		79 (56–91)	57 (46–67)	80 (58–92)	0.76	0.248
%PSA drop at 6 months		77 (67–84)	58 (44–79)	81 (59–91)	0.9	0.38
%PSA drop 12 months		61 (46–86)	48 (34–72)	70 (54–92)	0.7	0.112
PSA velocity 3 to 12 months (ng/ml/month)		0.14 (0.034–0.23)	0.044(-0.06-0.126)	-0.02(-0.1- 0.01)	0.005	0.001
PSA velocity linear regression (ng/ml/month)		0.14 (-0.009–0.32)	0.058 (0.015–0.17)	-0.002 (-0.09- 0.04)	0.032	0.007
% of patients with PSA at 12 months > nadir + 1		11/16 (69%)	6/18 (33%)	3/58 (5%)	< 0.001	< 0.001
Post ablation MRI	PIRADS > 3	8 (50%)	3 (16%)	3 (5.5%)	< 0.001	< 0.001
	PIRADS 3	0	4 (22%)	7 (12.7%)		
	No lesion	8 (50%)	11 (61%)	45 (81%)		

**Table 3:** Uni and multivariate logistic regression to identify predictors for clinically significant recurrence based on preoperative and postoperative variables, including PSA kinetics and post ablation MRI

	univariable		multivariable	
	OR (95% CI)	P	OR (95% CI)	P
Age	0.997 (0.94–1.057)	0.911	-	-
Baseline PSA	1.087 (1.002–1.178)	0.045	-	-
PSA density	11.51 (0.49–266)	0.127	-	-
NCCN group				
High + unfav int vs. low + fav int	6.181 (2.049-18)	0.001	5.9 (1.2–28)	0.027
Nadir PSA (continuous variable)	1.434 (1.079–1.906)	0.013	-	-
Nadir > 1 (yes vs. no)	9.97 (1.25–79.2)	0.03	-	-
Nadir > 2 (yes vs. no)	1.68 (0.57–4.9)	0.34	-	-
PSA at 12 months > nadir + 1 (yes vs. no)	14 (4.34-48)	< 0.001	7.2 (1.5–34)	0.012
PSA drop 3 months (continuous)	1.006 (0.98–1.028)	0.55	-	-
PSA drop 6 months (continuous)	0.99 (0.97–1.02)	0.949	-	-
PSA drop 12 months (continuous)	0.995 (0.96–1.023)	0.72	-	-
PIRADS at follow up MRI				
PIRADS 4–5 vs. none	10 (2.75-36)	< 0.001	8.2 (1.7–46)	0.009

NCCN groups were dichotomised into two groups, first including high and unfavorable intermediate risk, second including low and favorable intermediate risk. MRI lesions were divided into two subgroups, suspicious lesions including PIRADS 4 and 5, and other lesions (no lesions, PIRADs 1–3)

**Table 4:** Diagnostic performance for detecting clinically significant cancer (GG > 1) using various follow-up strategies to initiate biopsy includes: strategy A: conduct a biopsy only if the PSA level in the first year exceeds nadir + 1. strategy B: perform a biopsy only if MRI findings indicate PIRADS > 3. strategy C: order an MRI only if the patient Meets the PSA criteria. If the MRI reveals a lesion with PIRADS > 3, proceed with a biopsy. strategy D: perform biopsy if patient Meets PSA criteria and/or MRI with PIRADS > 3

	A	B	C	D
	PSA 1st year > nadir + 1	MRI PIRADS > 3	PSA criteria (nadir + 1) followed by MRI	Either PSA criteria or MRI criteria
Sensitivity	69%	50%	31%	88%
Specificity	86%	89%	97%	78%
PPV	52%	50%	71%	46%
NPV	92%	89%	86%	96%
Accuracy	83%	82%	85%	80%
Recurrences missed	5% (5/92)	8% (8/92)	12% (11/92)	2% (2/92)
Biopsies avoided	77% (71/92)	82% (75/92)	91% (84/92)	66% (61/92)
Unnecessary biopsies avoided	71% (66/92)	73% (67/92)	79% (73/92)	64% (59/92)

## Discussion

One of the unmet needs in FT is establishing a standardized follow-up protocol, including the definition of biochemical recurrence and integration of non-invasive tools such as MRI. This study identifies associations between PSA velocity, PSA rise above nadir + 1, and MRI changes after HIFU with the presence of clinically significant recurrence. These findings are crucial not only for properly selecting patients at higher risk of recurrence and in need of a prostate biopsy but also for reducing the number of unnecessary biopsies on patients with low recurrence yield.

While initial studies relied on “for cause” biopsies triggered by PSA outcomes (Phoenix criteria) [5], current recommendations favor using a protocol biopsy within 12 months post-HIFU. Although PSA reduction has been indicative of ablation effectiveness in other studies, our study did not find an association between static PSA levels and recurrence. Stabile et al. demonstrated that achieving a minimum 50% decrease in PSA levels within approximately 5 months indicates effective treatment and correlates with a 20% likelihood of requiring additional radical treatment within 5 years. Huber et al. [12] showed that a definition of biochemical recurrence using PSA nadir + 1 ng/ml at first year follow-up predicts treatment failure. This definition was validated in our study, showing a stronger association with csPCa compared to the percentage of PSA reduction. However, interpretation of PSA after HIFU could be challenging mainly due to tissue inflammation and volume of treated prostate. The threshold PSA level triggering further evaluation remains uncertain. For this reason, using dynamic evaluation of PSA rather than static measurement could be a better indicator of recurrence. In our study, PSA velocity proved to be a strong indicator of recurrence, achieving an AUC of 86%. Previous studies have not explored the role of PSA velocity in the context of post-HIFU treatment.

MRI has the potential to be a reliable surveillance tool. However, MRI interpretation after HIFU could be challenging given the post-treatment effect, such as gland shrinkage and fibrosis [13]. A novel scoring system PI-FAB (Prostate imaging after Focal Ablation) has been proposed to standardize the assessment based on diffusion intensity and focal enhancement of residual lesions [14]. However, it has not been validated yet in prospective studies. Novel imaging techniques including PSMA PET seem appealing in treatment monitoring as it has been evaluated in comparing baseline and posttreatment SUV parameters [15].

Therefore, given the scarcity of reliable evidence, it’s a common practice to conduct post-treatment biopsies. It’s worth noting that while individual tests may lack accuracy, their combination could potentially offer improved performance, enhancing patient selection for biopsy while simultaneously reducing the number of unnecessary biopsies performed. In our study, MRI had an elevated specificity and negative predictive value for ruling out CsPca. Combining MRI with PSA criteria resulted in a better diagnostic performance, improved the NPV and reduced the risk of missing recurrences. On the other hand, using PSA criteria as a triage test before MRI would improve specificity at the cost of missing more recurrences. In our opinion, both PSA and MRI should be included in the evaluation of treatment response within the first year.

Our study has several limitations. Although the follow-up protocol included a mandatory biopsy, some patients refused biopsy, given their stable and low PSA. In our study, the majority of these patients had a PSA less than 1ng/ml and a negative MRI and therefore were more likely to have a negative biopsy. In addition, the cohort was not homogeneous since some patients had concomitant or posterior BPH related surgery or the treatment modality (focal HIFU or hemi-ablation) was different. This could potentially alter the interpretability of PSA kinetics, mainly the reduction of PSA from baseline, as demonstrated in previous study from our group, the change in PSA during follow up varied if a BPH procedure was combined to focal therapy [16]. Finally, the definition of recurrence was based on overall recurrence rather than infield-treatment related-recurrence. The biological distinctions between HIFU-recurrent prostate cancer and de novo prostate cancer in terms of PSA production and MRI visibility remain unanswered.

## Conclusion

While surveillance biopsies post-HIFU are currently recommended to all patients, they are invasive and may lead to unnecessary procedures for patients with a lower risk of recurrence. Identifying those at higher risk of recurrence is crucial. In this study, we demonstrate that integrating PSA dynamics post-HIFU with MRI enhances patient selection for surveillance biopsies by effectively balancing the detection of recurrences while minimizing the risk of missing recurrences.

## Data Availability

No datasets were generated or analysed during the current study.
